# Which population groups are more likely to get too little sleep? Results of the ‘German Health Update’ (GEDA 2023)

**DOI:** 10.25646/14309

**Published:** 2026-08-19

**Authors:** Natali Kaufholdová, Almut Richter, Anke-Christine Saß, Anja Schienkiewitz

**Affiliations:** 1 Robert Koch Institute, Department of Epidemiology and Health Monitoring, Berlin, Germany; 2 Institute for Medical Information Processing, Biometrics and Epidemiology – IBE, LMU Munich, Germany; 3 Pettenkofer School of Public Health, Munich, Germany

**Keywords:** Prevalence, Sleep duration, Sleep deprivation, Weekends, Weekdays, Adults, Public health, Telephone survey, Germany

## Abstract

**Background:**

Short sleep duration is associated with an increased health risk.

**Methods:**

The data are based on a Germany-wide, representative telephone survey of adults conducted as part of the Health Monitoring Programme (GEDA). A sleep duration of less than seven hours per night (over the past four weeks) is defined as ‘short’.

**Results:**

Short sleep duration is observed on weekdays among 39.6 % of the adult population. Men are more likely than women to have short sleep duration, particularly men aged 45 to 64 years (53.0 %). At weekends, the proportion is significantly lower, particularly among 18- to 29-year-olds. Among those aged 65 years and older, however, there are barely any differences between weekdays and weekends.

**Conclusions:**

A significant proportion of the population experiences short sleep duration, which underlines the high public health relevance of this issue. Addressing this requires individual and societal preventive measures to promote healthy sleep patterns.

**Key messages**

► The proportion of people who sleep for less than seven hours per night is high in Germany: during the week, it is 39.6 %, and at weekends 22.7 %.
► The proportion of men with short sleep duration is higher than that of women.
► On weekdays, short sleep durations are more common across all age groups under 65.
► Among adults aged 65 and older, the proportion of people with short sleep duration does not differ between weekdays and weekends.

**Infobox GEDA 2023**

German Health Update (data collection 2023)

Data holder: Robert Koch Institute

Objectives: Provision of reliable information on the health status, health behaviour and health care of the population living in Germany

Study design: Cross-sectional telephone survey

Population: German-speaking population aged 18 years and older living in private households that can be reached via landline or mobile phone

Sampling: Random sample of landline and mobile telephone numbers (dual-frame method) from the ADM sampling system (Arbeitskreis Deutscher Markt- und Sozialforschungsinstitute e. V.)

Sample size: Ca. 1,000 participants per questionnaire module per month

Data collection period: January 2023 – February 2024

GEDA survey waves: GEDA 2009, GEDA 2010, GEDA 2012, GEDA 2014/2015-EHIS, GEDA 2019/2020-EHIS, Continuous survey between 2021 and 2023

Further information is available at www.rki.de/geda-en

## 1. Introduction

Due to its diverse physiological and regulatory functions, sleep is a fundamental prerequisite for maintaining health and human life [1]. International sleep recommendations define a sleep duration of less than seven hours in adults as short [2]. If a short sleep duration persists over a longer period and no functional disorder is present, the term ‘sleep deprivation’ is also used. 

A short sleep duration can lead to changes in hormonal regulation, the sympathetic nervous system and the circadian rhythm. Adults who sleep for less than seven hours exercise less and spend more time sitting [3]. Review articles suggest that short sleep duration is associated with high blood pressure [4, 5] and represents a significant risk factor for numerous non-communicable diseases [6, 7], such as obesity [8], diabetes mellitus [9] and cardiovascular diseases [5, 10]. Furthermore, there are implications for mental health: short sleep duration is an independent risk factor for the development of anxiety and depression [11]. At the same time, people with depression are highly likely to experience short sleep duration [12]. Sleep apnoea also frequently causes sleep interruptions, disrupting sleep continuity and potentially reducing effective sleep duration [13]. A lack of sleep, in turn, has an impact on road safety, for example through road traffic accidents caused by fatigue [14]. It also incurs economic costs due to reduced labour productivity caused by sleep deprivation and sickness-related absences from work [15]. 

Given the importance of sufficient sleep duration for both the individual and society, this article describes the proportion of the population who, according to self-reported data, sleep for less than seven hours and thus do not meet the recommendations for healthy sleep [2], broken down by weekdays and weekends and by gender, age and educational groups. 

## 2. Methods

The ‘German Health Update’ (GEDA) is a telephone-based cross-sectional survey of the resident population living in Germany, aimed at describing health status, healthcare provision and health behaviour, as well as identifying demographic and socio-economic factors [16]. The data on sleep behaviour is based on self-reported information regarding usual bedtime, time taken to fall asleep and morning wake-up time on weekdays and at weekends over the past four weeks. The survey was conducted as part of GEDA 2023 (Infobox) between May 2023 and February 2024. The analyses are based on data from 8,026 adults aged 18 and over. First, the actual duration of sleep was calculated as bedtime plus the time taken to fall asleep minus the time of waking. Participants with missing data for weekdays or weekends (n = 622), as well as those with an actual usual sleep duration of less than three hours or more than 13 hours (n = 55), were excluded from the analyses. Short sleep duration was operationalised in accordance with the National Sleep Foundation’s international definition as sleep lasting less than seven hours (< 7 h) [2], and is reported in this paper as the proportion of people who sleep for less than seven hours and thus do not meet the recommended amount of sleep.

For the analysis by gender, this study used respondents’ self-reported gender identity [17]. Individuals who did not identify as female or male were excluded from the analysis due to the small sample size (n = 35). Respondents were divided into four age groups: 18 to 29 years, 30 to 44 years, 45 to 64 years and 65 years and older. Educational groups were categorised as low, medium or high using the CASMIN classification (Comparative Analysis of Social Mobility in Industrial Nations) [18]. 

The analyses were carried out separately for weekdays and weekends. A weighting factor was applied to correct for deviations within the study sample from the German population in terms of gender, age, federal state (as at 31 December 2020) and educational groups [19]. This paper describes prevalences with 95 % confidence intervals (95 % CI). Differences between population groups were determined using the Wald test. P-values < 0.05 were considered statistically significant and are reported in the text as differences between groups. Individual values are not listed in the tables. The results were calculated using the statistical software packages R (version 4.3.0) and SAS 9.4 (SAS Institute, Cary, NC) with survey procedures for complex samples. 

## 3. Results

Among adults in Germany, 39.6 % sleep less than seven hours on weekdays and 22.7 % on weekends. The average duration of sleep is 05:58 hours on weekdays (95 % CI: 05:55 – 06:01) and 06:01 hours at weekends (95 % CI: 05:57 – 06:05). 

Table 1 shows the proportion of people who sleep for less than seven hours, by gender, age and educational groups. For both women and men, a higher proportion of people with short sleep durations is observed on weekdays compared with at weekends. Overall, this proportion is significantly higher among men than women, both on weekdays (women: 36.2 %, men: 43.2 %) and at weekends (women: 20.0 %, men: 25.5 %). 

On weekdays, short sleep duration was more prevalent among those aged 30 to 44 and 45 to 64 years than among those aged 18 to 29 years or 65 and older. Men aged 45 to 64 showed the highest prevalence, with one in two (53.0 %) sleeping fewer than seven hours. In adults aged 65 and older, the prevalence was significantly lower than in younger age groups. On weekdays, the proportion of people with short sleep durations tends to be higher in the low and medium educational groups than in the high educational group, although this difference is statistically significant only among men: 35.2 % of men with short sleep durations are in the high educational group, compared with 43.3 % in the low educational group and 46.1 % in the medium educational group. 

In the under-65 age groups, short sleep duration is significantly less prevalent at weekends than on weekdays. Compared with weekdays, this proportion is almost halved at weekends. The proportion of women and men with short sleep duration at weekends is lowest in the 18 to 29 age group (12.0 % of women and 16.6 % of men). In the 65 and older age group, there are no differences between weekdays and weekends. There are also no differences between educational groups. 

## 4. Discussion

These findings, based on participants’ self-reported sleep durations, show that a significant proportion of the population – up to 40 % on weekdays – usually sleeps for less than seven hours. This applies above all to men and, particularly on weekdays, to those aged under 65 years. 

There are only a few epidemiological studies in Germany that describe sleeping patterns in the population. The results of a nationwide online survey of around 2,000 adults in September/October 2024 showed that one third of the population sleeps six hours or less per night on weekdays, whilst at the weekend the figure is still one fifth [20]. In 2017, a survey commissioned by the Techniker Krankenkasse found that 32 % reported sleeping exactly 7 hours per night, although no distinction was made between weekdays and weekends [21]. A representative population survey in New Zealand (2014 – 2016) also found that 37.0 % of respondents slept for less than seven hours [22]. Significant differences between weekdays and weekends, with longer sleep duration at the weekend, have long been described in the literature [23]. Shorter sleep durations on weekdays are compensated for in the short term by longer sleep at the weekend, a phenomenon particularly observed among younger adults. However, this is no substitute for sufficient, regular sleep and does not compensate for all the biological consequences [24].

Gender differences based on wearable data show that, on average, women sleep longer than men [25]; men also report shorter sleep durations more frequently [26]. The available analyses show that shorter sleep durations are more common among men than women, both on weekdays and at weekends. Although the gender differences in sleep duration are small, it remains unclear whether these can be explained by women’s greater health awareness, or whether they are attributable to women’s greater need for sleep or other occupational and social factors. 

From the age of 65 onwards, there are no differences in sleep duration between weekdays and weekends compared to younger age groups [25]. Sleep duration is influenced not only by biological ageing processes and existing medical conditions [27], but also by living conditions in retirement, a phase of life without regular paid work, during which differences between weekdays and weekends become less pronounced. This study was unable to demonstrate marked differences based on educational attainment; however, a prospective analysis of the Socio-Economic Panel shows that a high socio-economic status is associated with optimal sleep duration, whilst a lower socio-economic status is associated with shorter sleep duration [28]. 

To put the results into context, it should be noted that sleep duration was calculated based on self-reported bedtime, time taken to fall asleep and morning wake-up time. In order to record the population’s sleep duration more precisely, the use of measuring devices, such as wearables, should be investigated. The threshold of less than seven hours’ sleep chosen for this study was derived from international sleep recommendations [2]. It should be noted that sleep durations of 6 to 10 hours for those aged 26 to 64 and 6 to 9 hours for those aged 65 and older may also be appropriate. Further analyses on this topic are to follow. Overall, sleep duration is only one dimension of sleep behaviour. Other aspects, such as sleep quality, daytime sleepiness, sleep disorders or sleep interruptions, could not be taken into account in this analysis, but are also important for determining adequate rest and associated health risks. Recent analyses show, for example, that women and people from lower educational groups are particularly affected by difficulties falling asleep and staying asleep [29].

As short sleep duration is associated with a significant risk of non-communicable diseases [6], and a considerable proportion of the population experiences it, this issue warrants closer examination in a public health context. Although not all people with short sleep durations face increased health risks, promoting healthy sleep patterns within the population requires a comprehensive approach. It is important to address not only individual preventive measures relating to sleep habits, including regular bedtimes and the sleeping environment, but also structural determinants, such as night-time noise pollution, domestic environmental factors (temperature and light exposure), and night or shift work. 

## Figures and Tables

**Table 1:** Proportion of people who sleep for less than seven hours by gender, age and educational group (n = 3,878 women, n = 3,436 men). Source: GEDA 2023

**Weekdays**					
****	**%**	****(** **95** ** % CI)****			
Total	39.6	(37.8 – 41.4)			
**Gender**					
Women (total)	36.2	(33.8 – 38.7)	Men (total)	43.2	(40.6 – 45.9)
**Women**			**Men**		
**Age groups**					
18 – 29 years	32.7	(25.3 – 41.1)	18 – 29 years	42.0	(35.1 – 49.1)
30 – 44 years	38.5	(33.0 – 44.3)	30 – 44 years	43.2	(37.3 – 49.4)
45 – 64 years	42.1	(38.4 – 46.0)	45 – 64 years	53.0	(48.6 – 57.4)
≥ 65 years	28.9	(25.4 – 32.6)	≥ 65 years	30.0	(26.0 – 34.2)
**Educational groups**					
Low	35.6	(30.1 – 41.4)	Low	43.3	(37.2 – 49.6)
Medium	37.3	(34.1 – 40.6)	Medium	46.1	(42.3 – 50.0)
High	32.5	(29.0 – 36.2)	High	35.2	(32.3 – 38.3)
**Weekend**					
****	**%**	****(** **95** ** % CI)****			
Total	22.7	(21.2 – 24.2)			
**Gender**					
Women (total)	20.0	(18.2 – 22.0)	Men (total)	25.5	(23.2 – 27.9)
**Women**			**Men**		
**Age groups**					
18 – 29 years	12.0	(7.5 –18.7)	18 – 29 years	16.6	(12.2 – 22.2)
30 – 44 years	17.2	(13.3 – 22.1)	30 – 44 years	22.8	(18.2 – 28.1)
45 – 64 years	21.1	(18.3 – 24.1)	45 – 64 years	30.8	(26.6 – 35.3)
≥ 65 years	25.5	(22.2 – 29.0)	≥ 65 years	26.4	(22.6 – 30.5)
**Educational groups**					
Low	22.2	(18.1 – 27.0)	Low	29.0	(23.7 – 34.9)
Medium	19.8	(17.4 – 22.5)	Medium	25.5	(22.2 – 29.0)
High	17.3	(14.6 – 20.4)	High	22.2	(19.6 – 25.1)

CI = confidence interval
